# Delayed Discovery and Repair of a Traumatic Arteriovenous Fistula (AVF) 58 Years After a Combat Explosion

**DOI:** 10.7759/cureus.97265

**Published:** 2025-11-19

**Authors:** Ravneet Nagra, Michael F Kanan, Jesse Martin, Radu Serban, Xavier R Packianathan

**Affiliations:** 1 Interventional Radiology, Saint Louis University School of Medicine, St. Louis, USA

**Keywords:** amplatzer plug, arteiovenous fistula, avf, interventional radiology, traumatic avf

## Abstract

An acquired arteriovenous fistula (AVF) is an abnormal connection between the arterial and venous systems, with delayed clinical presentation being rare. We present a 79-year-old male patient who was incidentally found to have a right iliac arteriovenous fistula (AVF) during imaging workup concerning for appendicitis, which would have been a rare diagnosis for a patient in this age group. He denied symptoms suggestive of a fistula and had a normal physical exam, making this an unusual asymptomatic presentation discovered 58 years after a combat blast injury. Endovascular repair required through-and-through vascular access and the deployment of an Amplatzer vascular plug for complete occlusion. Follow-up imaging confirmed decreased venous filling of the AVF, and the patient remains complication-free. This case highlights the need to be vigilant in patients with a history of remote trauma and the effectiveness of endovascular management for large AVFs.

## Introduction

An acquired arteriovenous fistula (AVF) is an abnormal vascular connection between a high-pressure arterial and low-pressure venous system. A majority of traumatic AVFs present after penetrating trauma. Traumatic AVFs create a low-resistance arteriovenous shunt, causing venous hypertension and potential distal hypoperfusion. Traumatic AVFs commonly present with symptoms such as pain, thrill, mass, rupture, or high output cardiac failure, with only 15% of patients presenting with no physical exam findings [1]. On average, patients with AVFs receive treatment within 2.3 years of the inciting trauma, with the longest time to treatment being 52 years [1,2]. We present a case of a patient incidentally found to have a traumatic AVF 58 years following a blast injury that occurred while in combat. Institutional review board (IRB) approval was not required for this report.

## Case presentation

**Figure 1:**
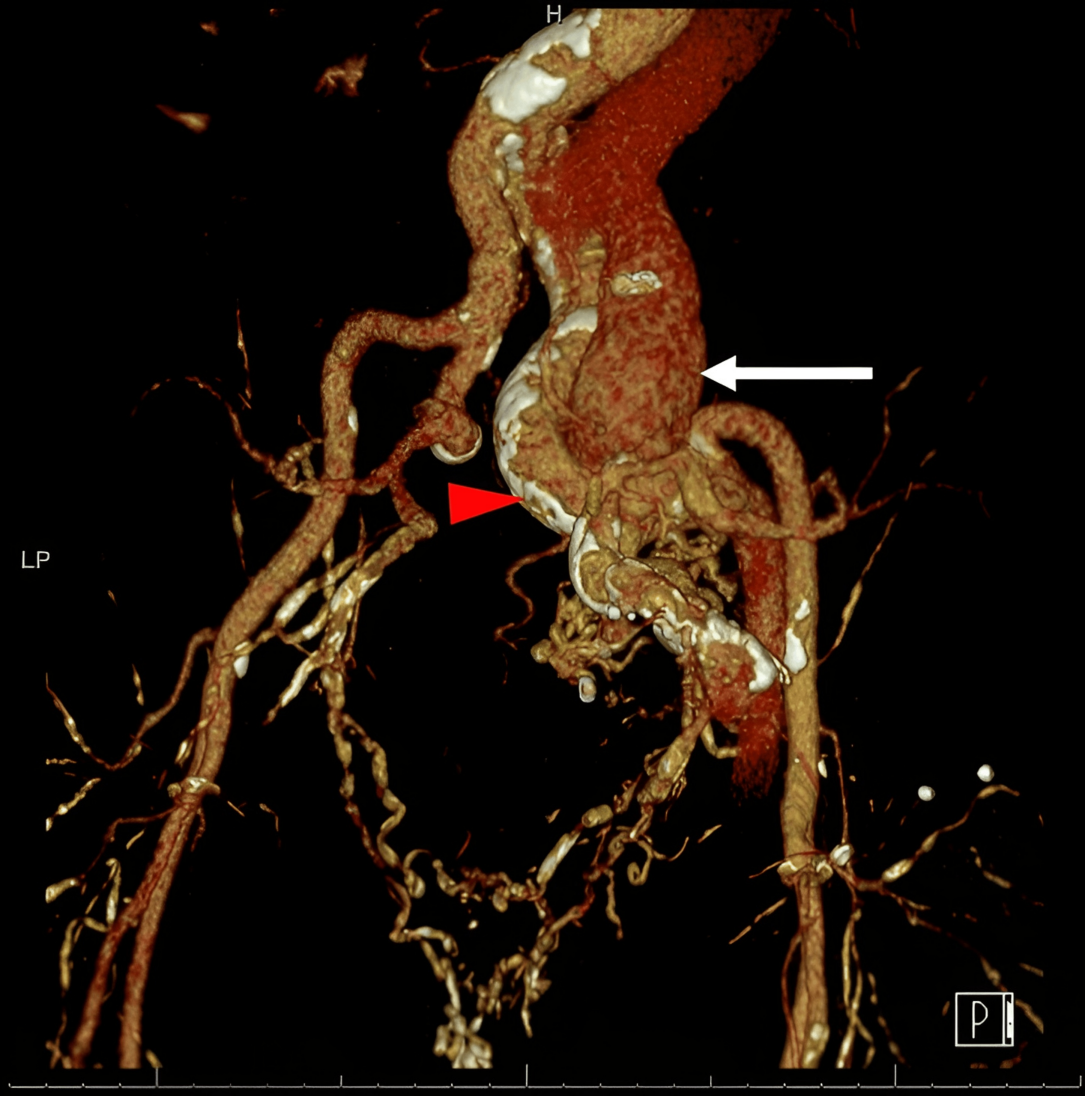
Three-dimensional CTA reconstruction of the arteriovenous fistula reveals marked calcification of the right internal iliac artery (IIA) (red arrowhead). The dilated right common iliac vein (CIV) (white arrow) is seen on this arterial phase due to early venous drainage, while the left CIV is not seen. Multiple overlapping dilated branches of the IIA are seen over the fistula. CTA: Computed tomography angiography. The 3D reconstruction using a Siemens Syndo, via the workstation at the Veterans Association (VA). All VA products are licensed (by paying a fee), and the reconstruction was done by Dr. Radu Serban.

A 79-year-old male patient with a history of atrial fibrillation, aortic valve replacement, type 2 diabetes mellitus, chronic heart failure with preserved ejection fraction (HFpEF), and restrictive cardiomyopathy was incidentally found to have a right iliac fistula along with foreign bodies resembling a shrapnel on a computed tomography (CT) abdomen and pelvis during appendicitis workup (Figure 1). The patient had a remote history of a shrapnel combat injury while serving in the Vietnam War, and no history of injury or surgery in this location, suggesting the blast injury was the cause of the AVF. During vascular assessment, the patient denied any AVF-related symptoms in his right leg, including claudication, a palpable mass, and his vascular exam was nonfocal.

**Figure 2:**
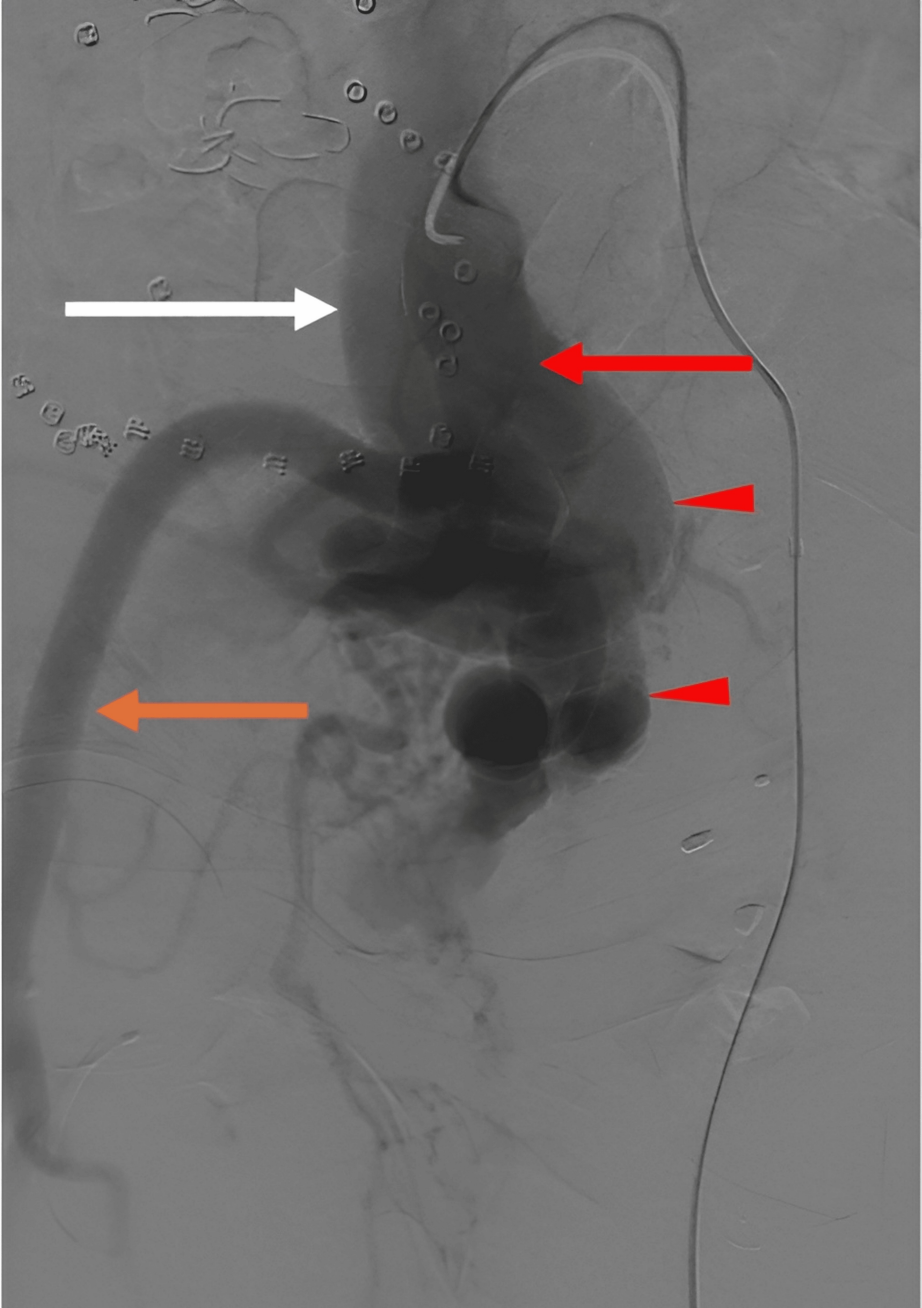
Digital subtraction angiography (DSA) shows a right common iliac artery (CIA) (red arrow) with early venous opacification of the common iliac vein (CIV) (white arrow) and marked tortuosity of the internal iliac artery (IIA) branches (red arrowheads), consistent with chronic high-flow changes. The external iliac artery is also seen (orange arrow).

**Figure 3:**
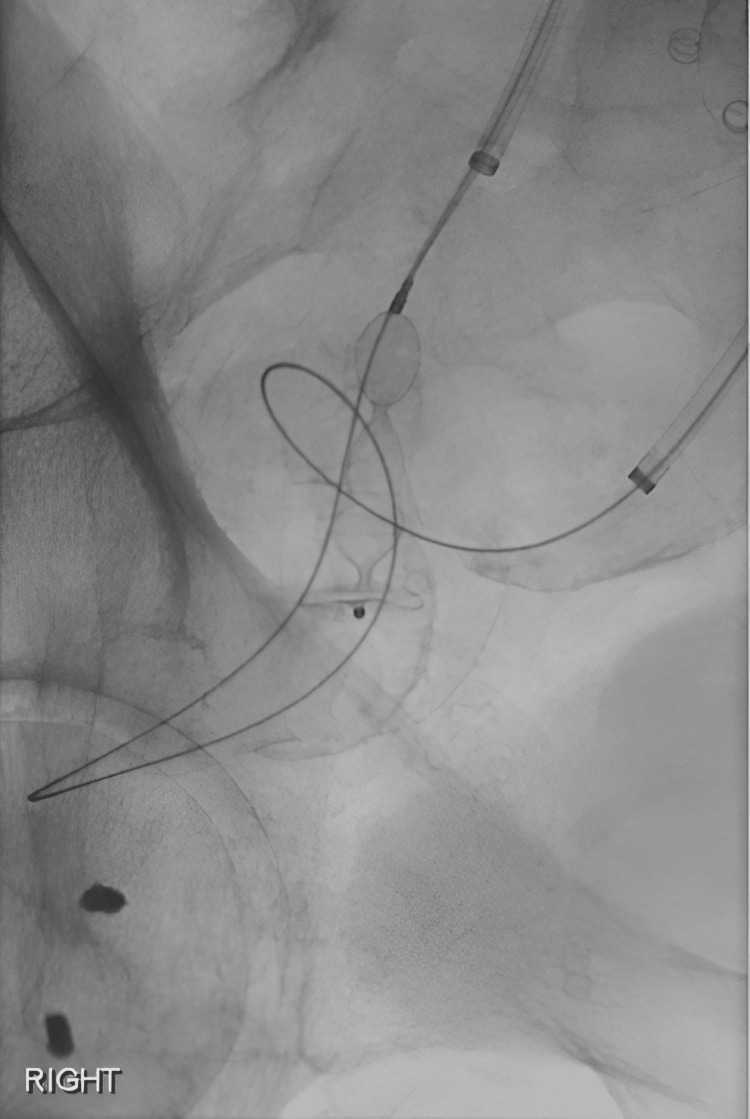
A 12 mm plug (Amplatzer Plug; St Jude Medical, Saint Paul, MN) was delivered through the sheath in the right IJ vein and deployed across the fistulous connection.

**Figure 4:**
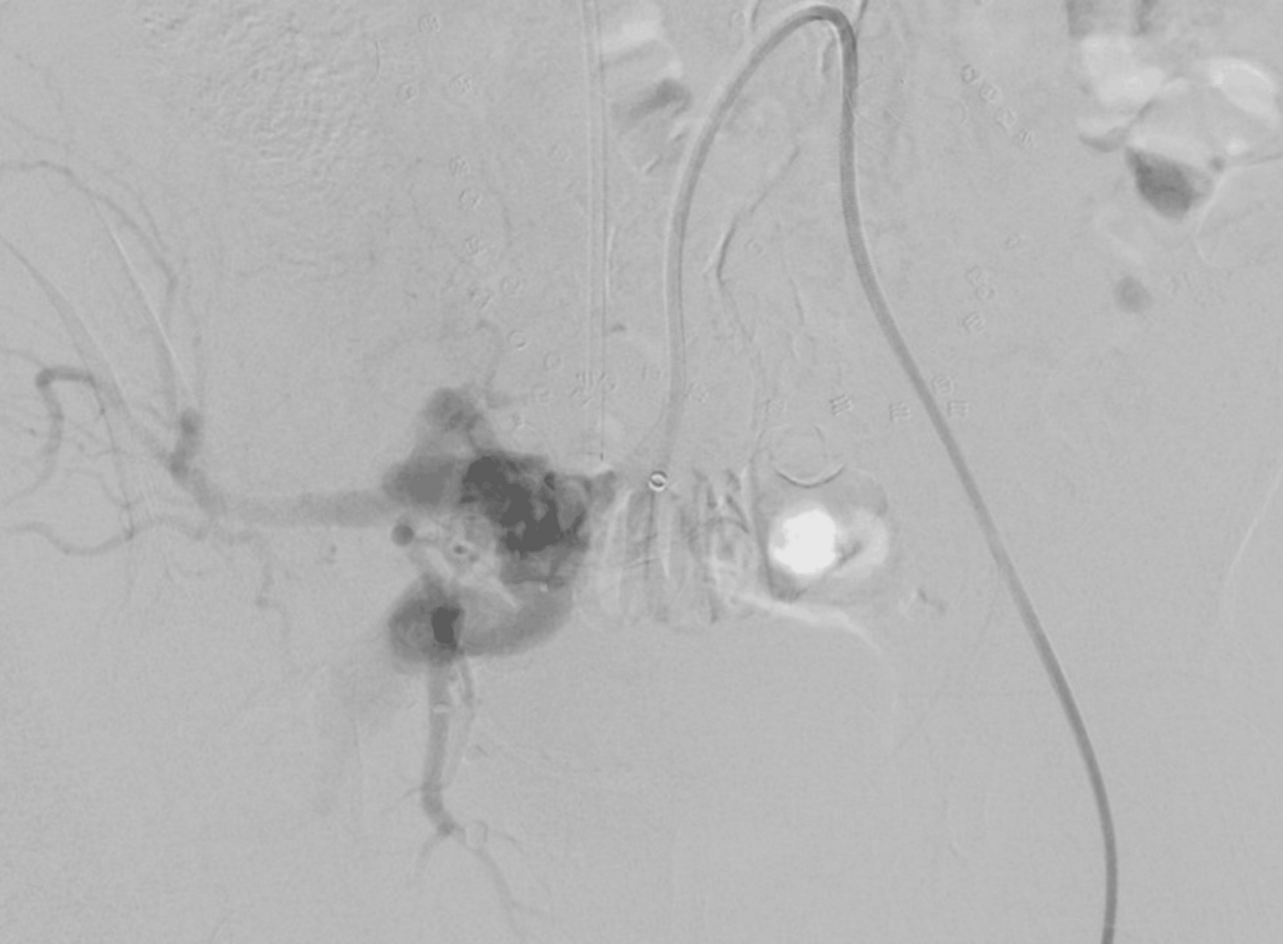
The post-intervention right IIA angiogram shows complete occlusion of the fistula without residual early venous drainage.

**Figure 5:**
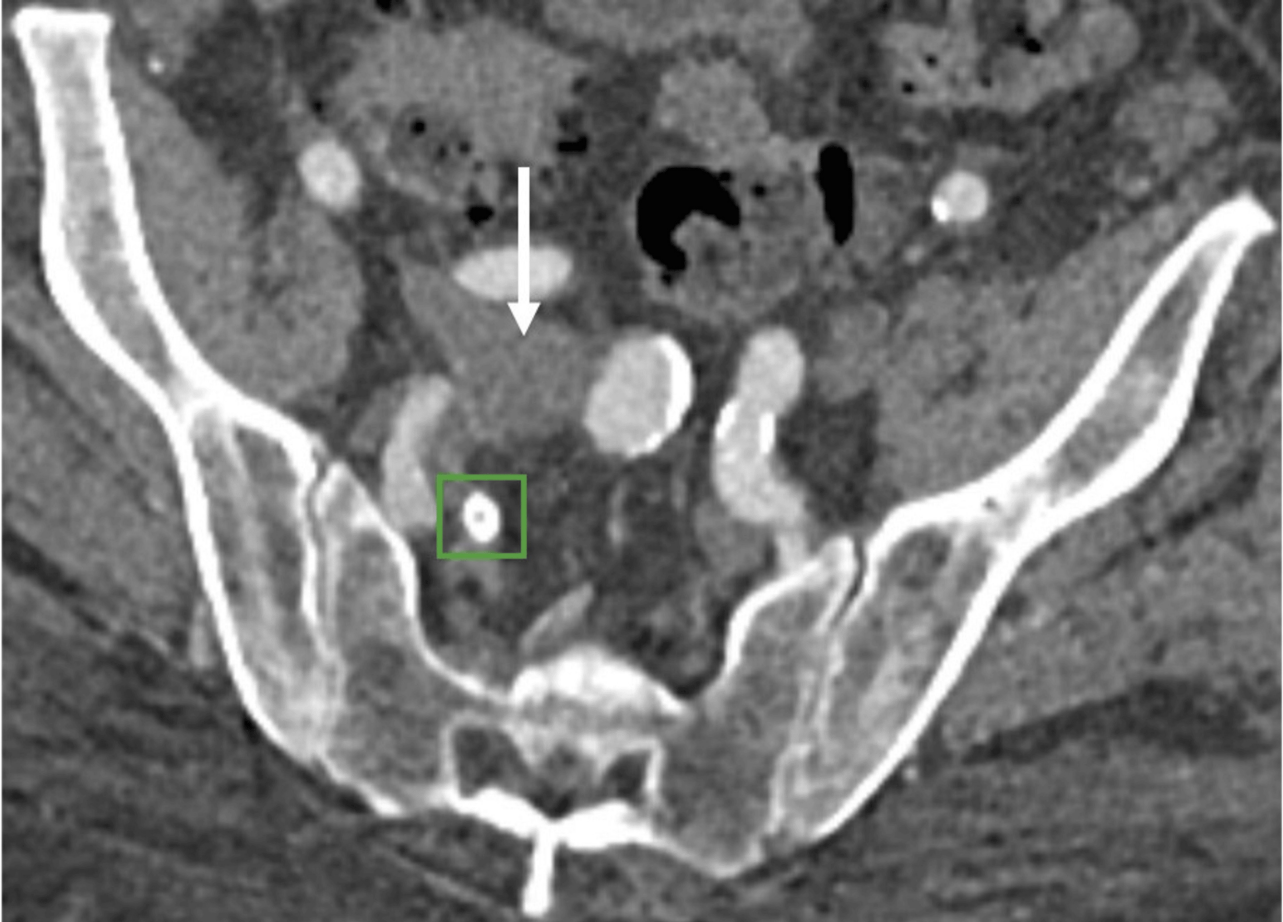
Post-embolization follow-up CTA abdomen pelvis demonstrates decreased caliber of the right common iliac vein (CIV; white arrow), with the plug seen in the previous fistulous connection (green box). In this arterial phase image, no early filling of the CIV is seen in comparison to pre-embolization. CTA: Computed tomography angiography.

**Figure 6:**
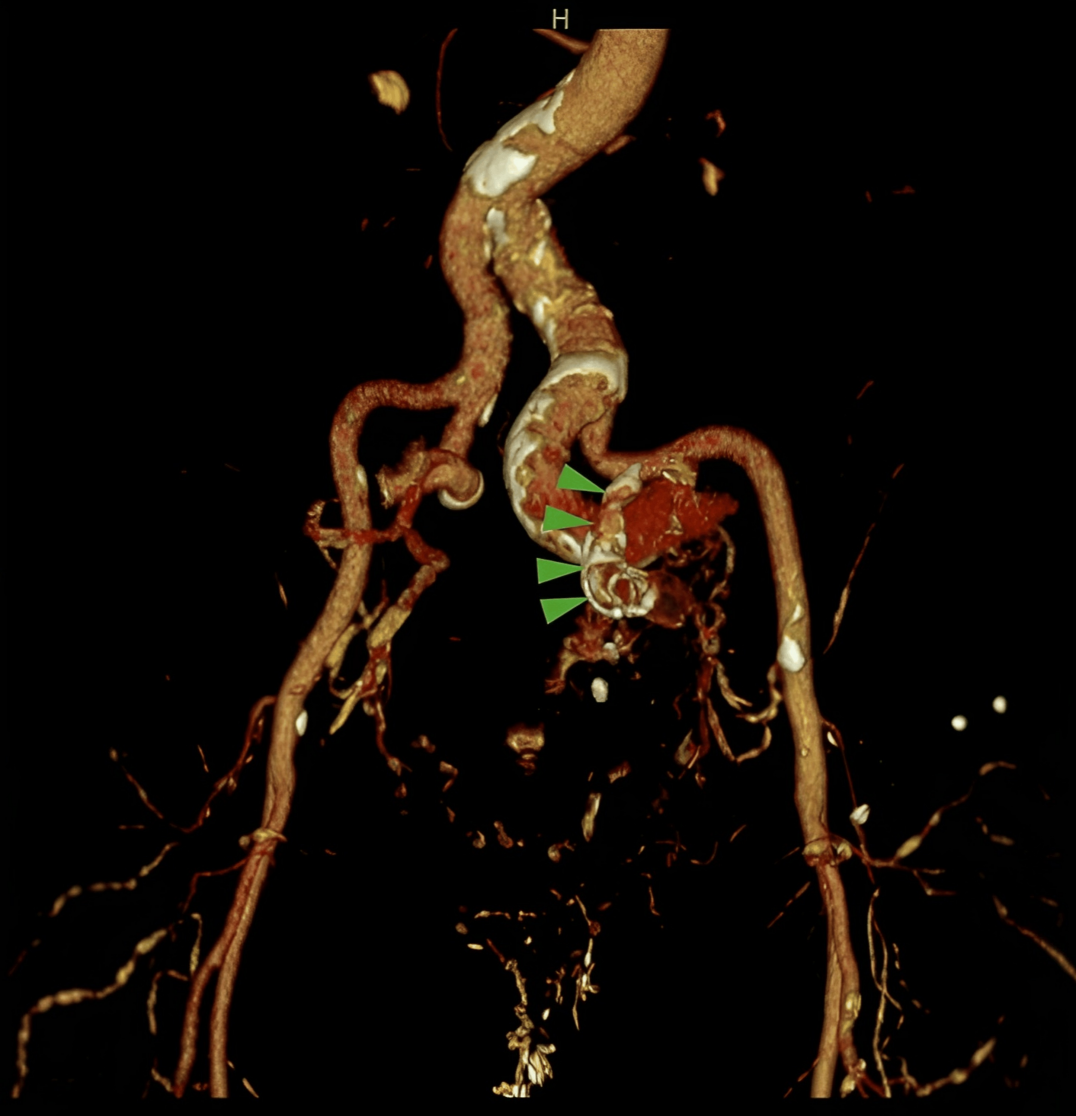
Three-dimensional CTA reconstruction demonstrates no right common iliac vein filling and thrombosis of a long segment of the right IIA proximal to the AVF plug. The location of the plug is demonstrated with green arrows. CTA: Computed tomography angiography. The 3D reconstruction using a Siemens Syndo, via the workstation at the Veterans Association (VA). All VA products are licensed (by paying a fee), and the reconstruction was done by Dr. Radu Serban.

For the AVF repair, access was first obtained from the right internal jugular (IJ) vein with a 90 cm 7 Fr sheath (Pinnacle Destination; Terumo, Tokyo, Japan). Using knowledge based on CT and anatomical landmarks, we attempted retrograde access of the fistula, which was unsuccessful. We then obtained left common femoral artery access and the initial angiogram from the right internal iliac artery demonstrated the known AVF between a branch of the right internal iliac artery and the right common iliac vein with marked tortuosity and dilation, making the exact branch of the right internal iliac artery difficult to determine (Figure 2). We exchanged for a 7 Fr 45 cm sheath (Pinnacle Destination) and placed it over the iliac bifurcation into the right internal iliac artery. Then, we navigated a 5 Fr catheter (Kumpe; Cook Medical, Bloomington, IN) and 0.035” wire (Glidewire, Terumo, Tokyo, Japan) through the fistula to the inferior vena cava (IVC). Using the IJ vein access, a trilobed snare (Atrieve Vascular; Argon Medical, Plano, TX) was used to snare the glide wire in the IVC and floss the AVF, with through-and-through access obtained from the right IJ to the left common femoral artery. We were then able to advance the sheath from the right IJ into the fistula, and the venous outflow tract measured 8 mm on angiogram. We then placed a 12 mm vascular plug (Amplatzer Plug; St Jude Medical, St. Paul, MN) into the AVF connection from the right IJ and embolized the communication to complete stasis (Figures 3, 4). This was confirmed on follow-up CT, which demonstrated a smaller common iliac vein (Figures 5, 6). At nine-month follow-up, the patient remains asymptomatic, without signs of decreased preload or deep venous thrombosis (DVT). Quantitative fistula diameter and pre- and post-cardiac function data were not available due to the case's retrospective nature and imaging constraints.

## Discussion

Traumatic AVFs can present in various ways depending on the mechanism and location of injury, as first described by William Hunter in 1761. Further, the characterization of traumatic AVFs varies between military personnel and civilians. Penetrating trauma due to gunshot wounds (GSW) or stab wounds accounts for the majority of civilian AVFs. In a series of civilian traumatic AVFs, approximately 63% were from stab wounds, 26% from GSWs, and around 1% were from blunt trauma [3]. In penetrating traumas, direct arterial injury and damage lead to blood extravasation, hematoma formation, and pseudoaneurysms. Concurrent vein damage leads to AVF formation. There are reports of AVF presentation years after trauma; however, in contrast to this case, those patients typically present with physical exam findings [1]. Delayed AVF presentation often results from the time required for tract maturation due to inflammation and angiogenesis [4,5]. A chronic AVF, as in this extreme case, leads to further changes, such as dilation and tortuosity of nearby vasculature. This can cause venous and lymphatic complications, with late manifestations being high-output HF, edema, and diminished distal perfusion [6,7]. Although the patient had HFpEF, his multiple cardiac comorbidities likely contributed to his HF, and he lacked classic high-output HF symptoms.

Treatment of AVFs is often through endovascular embolization with coils, plugs, or liquid embolic agents to occlude the fistula. It is atypical for AVFs to be managed conservatively due to progressive enlargement and complications. In a literature review of 210 traumatic AVFs, only 3.7% did not require treatment [1]. Current literature on traumatic AVF management is limited to case series and observational studies, with support for either monitoring for progression, or treating AVFs impacting named vessels due to their complications [8].

## Conclusions

Both open and endovascular repair of traumatic AVFs are viable options for treatment. Endovascular approaches can offer lower post-procedural complications and can be helpful in situations where a patient is unstable. In our case, given the AVF's large size, perceived rupture risk, anatomic suitability for endovascular treatment, and a strong patient preference, we opted to endovascularly treat this AVF. This is one of the first reported cases of an asymptomatic AVF presentation from a shrapnel blast explosion - found 58 years after the inciting event - and underscores the importance of remaining vigilant when assessing patients with remote histories of trauma.
